# Incidental renal cell carcinoma presenting in a renal transplant recipient with autosomal dominant polycystic kidney disease: a case report

**DOI:** 10.1186/1752-1947-6-154

**Published:** 2012-06-13

**Authors:** Toshihiro Misumi, Kentaro Ide, Takashi Onoe, Masataka Banshodani, Hirofumi Tazawa, Yoshifumi Teraoka, Ryuichi Hotta, Masahiro Yamashita, Hirotaka Tashiro, Hideki Ohdan

**Affiliations:** 1 Department of Surgery, Division of Frontier Medical Science, Programs for Biomedical Research, Graduate School of Biomedical Sciences, Hiroshima University, 1-2-3 Kasumi Minami-ku, Hiroshima, 734-8551, Japan

## Abstract

**Introduction:**

We report an instructive case of incidental renal cell carcinoma in a patient with autosomal dominant polycystic kidney disease who underwent simultaneous bilateral native nephrectomy and living donor renal transplantation.

**Case presentation:**

A 57-year-old Asian man with end-stage kidney disease due to autosomal dominant polycystic kidney disease received a living kidney graft from his brother. Because of recurrent infection, chronic pain and enlarged kidneys, he underwent a bilateral nephrectomy with concomitant renal transplantation. The total weight of the removed kidneys was 6kg; the maximal diameter of the larger kidney was 28cm. His left kidney had a 1cm diameter tumor. Pathology indicated papillary renal cell carcinoma. At the time of this report, the transplant kidney function was normal with no evidence of local recurrence or distant metastasis.

**Conclusion:**

This case shows and reinforces the importance of considering the possibility of an occult malignancy in the native kidneys of patients with autosomal dominant polycystic kidney disease. Simultaneous bilateral native nephrectomy should be considered in these renal transplant recipients not only for preventing the development of adverse symptoms but also for detecting an occult malignancy.

## Introduction

Autosomal dominant polycystic kidney disease (ADPKD) is one of the best-known genetic diseases. Almost half of patients with ADPKD will develop end-stage renal disease (ESRD) by the age of 60 [1]. According to a survey by the Japanese Society for Dialysis Therapy, approximately 3% of dialysis patients have ADPKD. The clinical course of ADPKD typically includes refractory abdominal discomfort and flank pain caused by renal enlargement, experienced by approximately 60% of patients [2]. The kidneys of patients with ADPKD usually continue to increase in size even after patients begin dialysis, and mass effects can lead to severe complications including recurrent cyst infections and urinary tract infections, severe hypertension, bleeding into the cysts, and intractable pain. At the time of transplantation, these nonfunctional kidneys are massively enlarged [3]. Therefore, pretransplant native nephrectomy is performed to create space in the pelvis, to decrease compression by the enlarged polycystic kidney, and to prevent development of various symptoms. In recent years, transcatheter arterial embolization (TAE) of the renal artery has been reported as a renal contraction therapy for ADPKD; however, the effects of this therapeutic method remain controversial.

Here, we present a case of incidental renal cell carcinoma (RCC) in a patient with ADPKD who underwent simultaneous bilateral native nephrectomy and living donor renal transplantation. This case is instructive in that the simultaneous bilateral native nephrectomy offered an opportunity to detect an occult malignancy in the resected native kidney of a renal transplant patient with ADPKD.

## Case presentation

**Figure 1:**
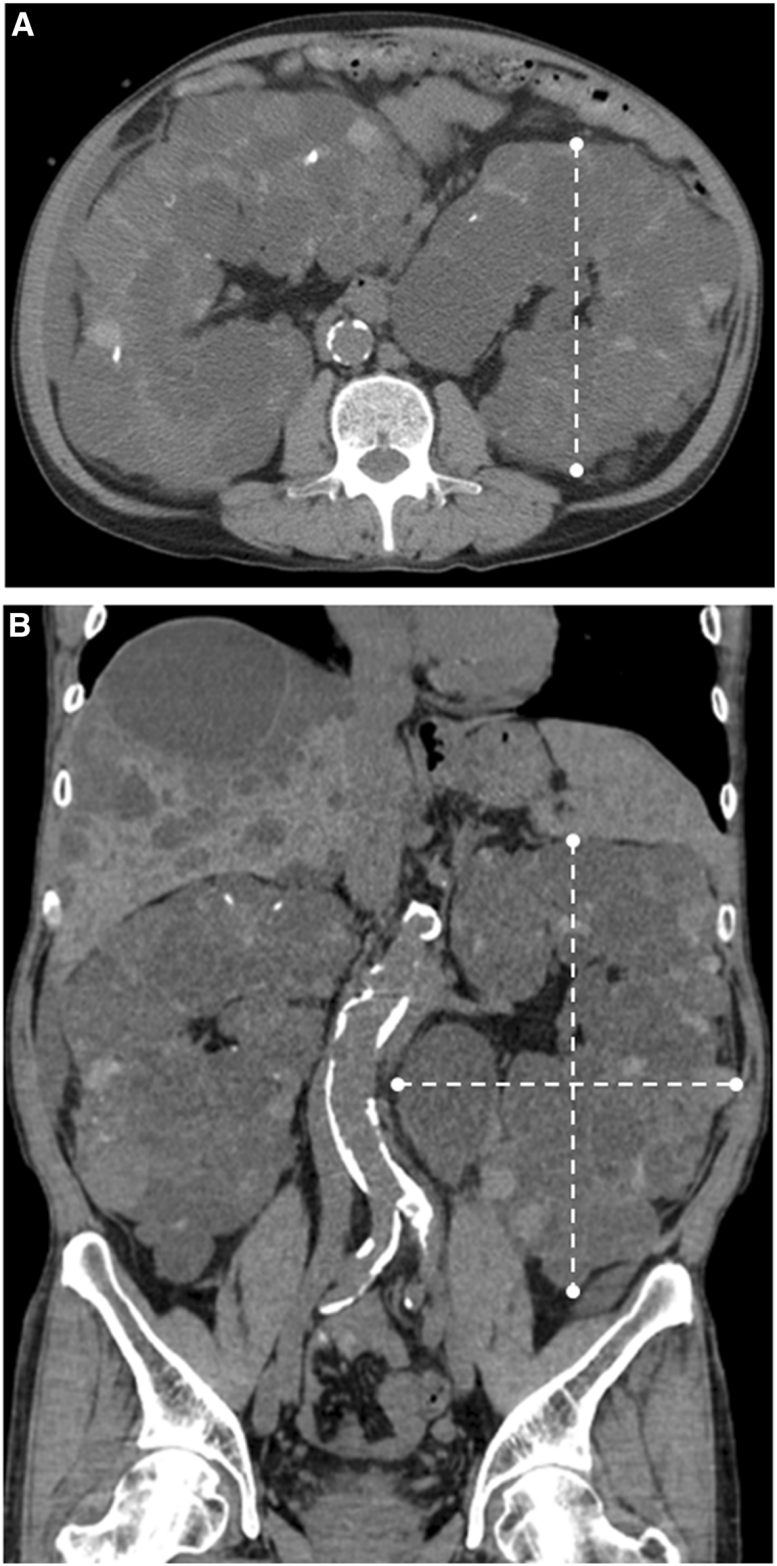
**Computed tomography showing innumerable cysts of variable size in both kidneys and the liver. (A)** Axial and **(B)** coronal unenhanced computed tomography showing enlarged kidneys and multiple liver cysts. The three greatest diameters of the kidney are indicated by dashed lines. No tumors were identified in other organs.

A 57-year-old Asian man had ESRD due to ADPKD. At the time of referral, he had undergone peritoneal dialysis for 7 years. Although he had recurrent cystic infection and chronic pain, his urine volume was maintained at about 300mL/day. A physical examination showed two obvious masses in his abdomen extending from his rib cage into his pelvis. Laboratory results were within normal ranges except for carcinoembryonic antigen (13.4ng/mL; normal range <5ng/mL) and carbohydrate antigen 19-9 (1,736U/mL; normal range <37U/mL). Computed tomography (CT) showed innumerable cysts of variable size in both kidneys and his liver (Figure 1A,B). Positron emission tomography-CT did not show increased uptake in either kidneys or in his other organs, and an endoscopic examination demonstrated the absence of tumors.

**Figure 2:**
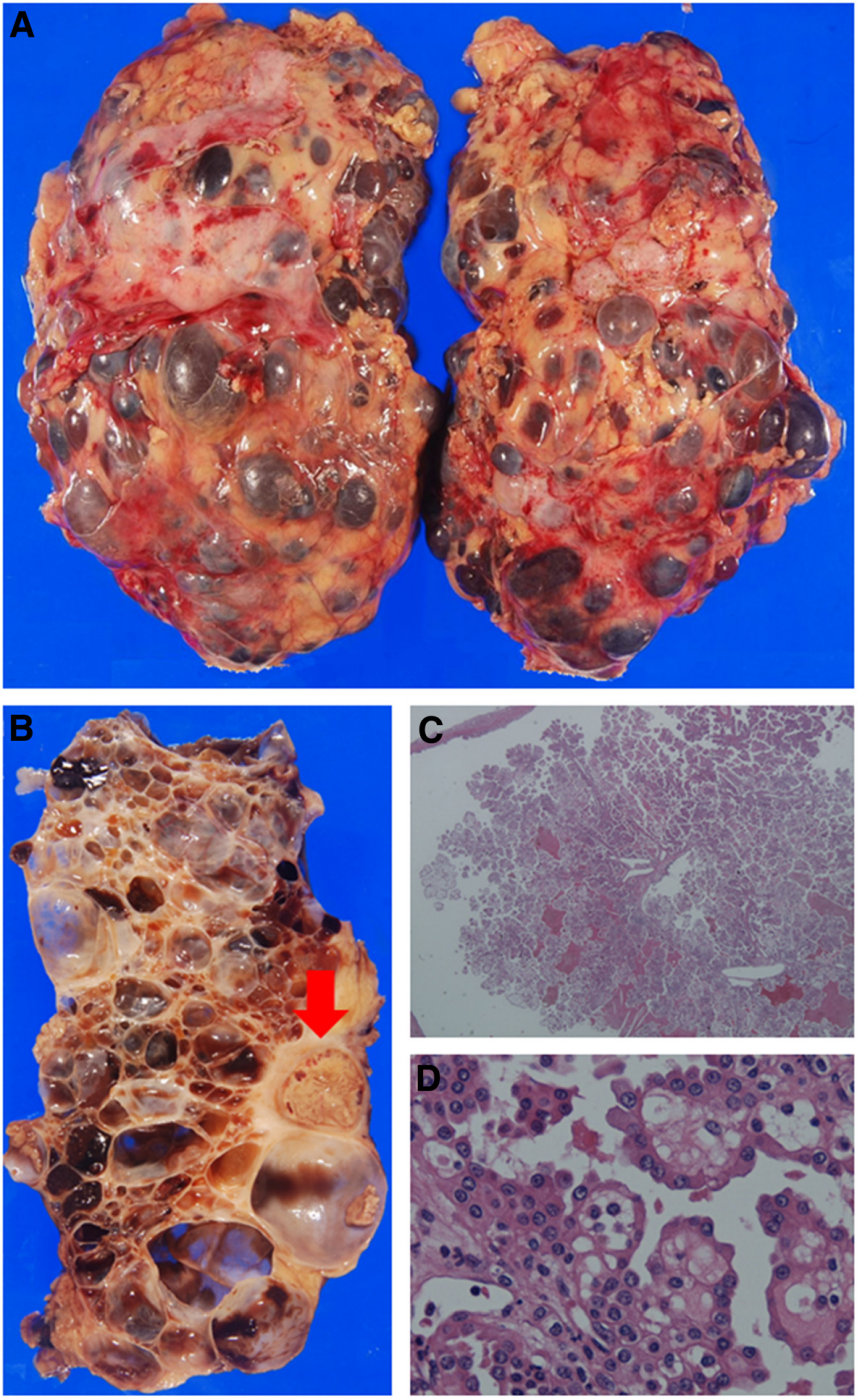
**Kidney specimens and tumor histopathology.** Kidney specimens from a patient who underwent simultaneous bilateral nephrectomy and histopathologic findings of the left kidney tumor. **(A)** Right kidney measured 26 × 16 × 13cm and left kidney measured 28 × 17 × 13cm. **(B)** Cross-section of the left kidney shows a slightly yellow solid tumor (arrow). **(C)** Hematoxylin-eosin stain, original magnification × 100 and **(D)** hematoxylin-eosin stain, original magnification × 400 show papillary renal cell carcinoma with large columnar cells with eosinophilic cytoplasm and edematous or fibrous thick stalks.

Because of the recurrent infection, chronic pain and enlarged kidneys, our patient underwent a bilateral nephrectomy with concomitant living donor renal transplantation. He received a renal graft from his brother (ABO blood group compatible, human leukocyte antigen full match, T-cell flow cytometry crossmatch assay negative). A bilateral nephrectomy was performed through an abdominal midline incision, and the renal allograft was placed in the right iliac fossa through a modified Gibson incision. The renal graft was positioned in his retroperitoneum to prevent renal pedicle torsion. The operative time was 533 minutes, including bilateral nephrectomy, removal of the continuous ambulatory peritoneal dialysis catheter, and the renal transplantation. The estimated blood loss was 2,240mL (including urine from the renal allograft and ascites). He had no surgical complications. The total removed kidney weight was 6kg (8.5% of his total body weight) (Figure 2A) and the maximal diameter of his larger left kidney was 28cm. There was a tumor in his left kidney, 1cm in diameter (Figure 2B).

The final pathological diagnosis was a type 2 papillary RCC, eosinophilic variant, pT1a (Figure 2C,D). The quadruple immunosuppression induction protocol comprised cyclosporine, mycophenolate mofetil, basiliximab and methylprednisolone. He showed an immediate improvement in renal function. His creatinine level decreased gradually and reached 0.59mg/dL on post-transplantation day 28. His postoperative recovery was uneventful, and our patient received regular follow-up care at our outpatient clinic. At the time of this report, the transplanted kidney function was normal with no evidence of local recurrence or distant metastasis.

## Discussion

Generally accepted indications for nephrectomy in ADPKD cases are recurrent pyelonephritis, cyst hemorrhage requiring repeat transfusions, pain refractory to medical management, and massively enlarged kidneys that cause early satiety or extend into the true pelvis [4]. An enlarged polycystic kidney can cause mechanical compression of the transplanted kidney and ureter with consequent disturbance of urine flow and vascular disorders [5]. Furthermore, there may be technical difficulties during transplantation.

Traditionally, operations concurrent with transplant recipient surgery have been avoided due to concerns that a complication of the additional surgery could endanger the transplant and that high dose induction immunosuppression could increase the complication risk of extra surgery. However, previous studies have demonstrated that pretransplant nephrectomy in patients with ADPKD results in an increased risk of perioperative morbidity, such as fluid overload, congestive heart failure, hyperkalemia, anemia, and renal osteodystrophy [6]. Staging nephrectomy and transplantation also requires an additional operation with anesthetic exposure and associated hospitalization. Recent studies show that concurrent nephrectomy with renal transplantation does not increase mortality or morbidity [7-9].

Approaches to ADPKD have included unilateral and bilateral native nephrectomy. Possible advantages of unilateral nephrectomy are the ability to avoid violating the peritoneum as well as a likely shorter operative time and decreased blood loss [4]. However, pyelonephritis associated with ADPKD can produce fatal sepsis in immunosuppressed patients. Bilateral nephrectomy, not unilateral nephrectomy, eliminates this risk in patients with recurrent infections. Bilateral nephrectomy also eliminates cyst hemorrhage and decreases the chronic pain associated with ADPKD that produces considerable patient morbidity. In our case, our patient had undergone peritoneal dialysis and his urine volume had been maintained, although he also suffered from recurrent cystic infection and chronic pain. Therefore, we performed simultaneous renal transplantation and bilateral nephrectomy because a hemodialysis bridge was unnecessary and the process eliminated the risk of recurrent infections.

Particular attention should be paid to exclude the presence of solid or cystic tumors within the kidneys or other abdominal organs. RCC is present in ADPKD at the same frequency as in the general population [10]. Recently, incidental detection of RCC has been increasing due to the development and expanded use of improved CT and ultrasound in hospitals [11]. Nevertheless, the preoperative diagnosis of small RCC in ADPKD is difficult because the tumor may be masked by the complex cystic background superimposed by bleeding, degenerated blood clots, proteinaceous debris, and infection, in addition to renal failure preventing intravenous contrast injection in many cases.

Patients who have been successfully treated for pretransplant malignancy are deemed suitable candidates for transplant. However, the precise waiting period should be determined on an individual basis by tumor type, staging, and response to therapy. Previous studies revealed that organ-confined RCCs, especially those <4cm in diameter (pT1a), could be completely cured by partial or radical nephrectomy but more enlarged RCCs (>7cm, T2) require an adequate disease-free waiting period after surgery [12,13]. Tumors >4cm in diameter can be detected with preoperative CT or ultrasound even if they exist in a background of ADPKD. Therefore, we believed that staging nephrectomy and transplantation may be suggested when RCC is suspected in patients with ADPKD. However, patients with ADPKD preoperatively diagnosed as having no malignancy might still have an occult malignancy in the native kidneys. Thus, simultaneous bilateral native nephrectomy could offer an advantageous opportunity to detect an occult malignancy in the resected native kidneys on histological study.

Recently, TAE of the renal artery has been reported as an effective and less invasive renal contraction therapy for ADPKD. The volume reduction obtained after TAE has reached 60% after 6 months [14]. Thus, TAE of enlarged polycystic kidneys has been proposed as an alternative to nephrectomy before renal transplantation. However, several problems must be addressed before the procedure is performed. Cornelis *et al*. reported that if kidney volume is excessive (>5000cm^3^), TAE must be considered carefully because of the high risk of an insufficient reduction in volume [15]. In our case, his baseline kidney volumes were calculated using the ellipsoid formula from the three greatest diameters (volume = length × depth × width × 0.5233) on unenhanced CT acquisition. His baseline right kidney volume was 5,364cm^3^ and his left was 5,387cm^3^. Furthermore, renal TAE therapy alone cannot cure a malignant renal tumor, because the capillary and small artery networks are well-developed in ADPKD [14]. Thus, delayed detection and treatment of RCC in ADPKD might be avoided if this therapeutic method were employed for the treatment of ADPKD in kidney transplant candidates.

## Conclusion

We present an instructive case of incidental RCC in a patient with ADPKD who underwent simultaneous bilateral native nephrectomy and living donor renal transplantation. This case shows and reinforces the importance of considering the possibility of an occult malignancy in the native kidneys of patients with ADPKD. Simultaneous bilateral native nephrectomy should be considered in these renal transplant recipients not only for preventing the development of adverse symptoms but also for detecting an occult malignancy.

## Consent

Written informed consent was obtained from the patient for publication of this case report and any accompanying images. A copy of the written consent is available for review by the Editor-in-Chief of this journal.

## Competing interests

The authors declare that they have no competing interests.

## Authors’ contributions

TM and KI assisted in the surgery, analyzed and interpreted the patient data and were major contributors in writing the manuscript. TO, YT, MB, HTaz, RH, MY, and HTas also assisted in the surgery. HO performed the surgery and was a major contributor in writing the manuscript. All authors read and approved the final manuscript.
